# DNA barcoding uncovers two putative new species in *Trachelyopterus* (Siluriformes: Auchenipteridae)

**DOI:** 10.1111/jfb.70540

**Published:** 2026-07-07

**Authors:** Fiorindo José Cerqueira, Maelin da Silva, Chrystian Aparecido Grillo Haerter, Sandra Mariotto, Liano Centofante, Eliana Feldberg, Orlando Moreira Filho, Vladimir Pavan Margarido, Daniel Rodrigues Blanco, Roberto Laridondo Lui

**Affiliations:** ^1^ Programa de Pós‐Graduação em Conservação e Manejo de Recursos Naturais Universidade Estadual do Oeste do Paraná Cascavel Brazil; ^2^ Programa de Pós‐Graduação em Biologia Evolutiva Universidade Estadual de Ponta Grossa Ponta Grossa Brazil; ^3^ Instituto Nacional de Pesquisas da Amazônia Coordenação de Biodiversidade, Laboratório de Genética Animal Manaus Brazil; ^4^ Instituto Federal de Educação, Ciência e Tecnologia de Mato Grosso Mato Grosso Brazil; ^5^ Universidade Federal do Mato Grosso Cuiabá Brasil; ^6^ Departamento de Genética e Evolução Universidade Federal de São Carlos São Carlos Brazil; ^7^ Universidade Tecnológica Federal do Paraná Campus Santa Helena Santa Helena Brazil

**Keywords:** Bayesian analysis, catfish, *COI*, mitochondrial DNA, neotropical

## Abstract

*Trachelyopterus* is a cryptic genus within the Neotropical catfish family Auchenipteridae with a complex history of taxonomic revisions. Despite the notable morphological and genetic similarities among its species, some studies suggest that the genus harbours greater diversity than is currently recognized, including additional species yet to be formally described. One of the most widely used and effective approaches for investigating species delimitation and assessing biodiversity is DNA barcoding. The application of DNA barcoding has expanded considerably over the past decade, contributing significantly to the understanding of diversity across numerous groups of Neotropical fishes. Thus, in this study, we employed the DNA barcoding approach in both phenetic and phylogenetic contexts to investigate species diversity within the genus *Trachelyopterus*. Our sampling included four valid species previously identified based on morphology, collected from six hydrographic basins across the Neotropical region: *Trachelyopterus galeatus*, *Trachelyopterus striatulus*, *Trachelyopterus porosus* and *Trachelyopterus coriaceus.* Our results corroborate the validity of these four species and reveal two distinct lineages: one from the Araguaia River basin and another from the Pantanal in the Paraguay River basin, both of which may represent previously unrecognized species.

## 1 INTRODUCTION

The Neotropical region harbours the greatest freshwater fish diversity on the planet (Albert & Reis, 2011; Dagosta & Albert, 2024; Tedesco et al., 2017). Recent estimates suggest that more than 9000 species occur in South America (Dagosta & de Pinna, 2019; Reis et al., 2016), exceeding the approximately 5160 species formally described for the continent (Dagosta & de Pinna, 2019; Reis et al., 2016). Although our understanding of fish biodiversity has increased over the last few years, significant knowledge gaps remain for the Neotropical region, particularly regarding the ecology, biology and phylogenetic relationships of many species (Calegari et al., 2019).

This diversity has traditionally been identified through analysis of morphological characters. However, DNA‐based studies have revealed considerable cryptic diversity and numerous undescribed species, particularly within groups in which morphological similarity hampers accurate identification (e.g., Cooke et al., 2012; Fernandes et al., 2005; Nagamachi et al., 2010). In addition, phenotypic plasticity may further contribute to misidentifications, while morphological identification keys are often applicable only to adult life stages (Hebert et al., 2003; Valentini et al., 2008). Beyond these limitations, incongruences among data generated by taxonomists, molecular biologists and geneticists are frequently reported. Such discrepancies can lead to taxonomic conflicts at different hierarchical levels, even within families that have been extensively studied, such as Auchenipteridae (e.g., Akama, 2004; Birindelli, 2014; Britski, 1972; Calegari et al., 2019; Curran, 1989; Ferraris, 1988; Mees, 1974; Royero, 1999).

The family Auchenipteridae is currently divided into two subfamilies: Centromochlinae and Auchenipterinae (Calegari et al., 2019). The latter includes the genus *Trachelyopterus*, which has been the focus of several studies addressing the taxonomic validity of the former genus ‘*Parauchenipterus*’, a long‐standing taxonomic controversy within the family (Akama, 2004; Curran, 1989; Mees, 1974; Royero, 1999). Throughout its taxonomic history, ‘*Parauchenipterus*’ has been treated as synonymous with other Auchenipteridae genera, including *Auchenipterus* (Günther, 1864), *Trachycorystes* (Britski, 1972; Eigenmann & Eigenmann, 1888, 1890; Regan, 1911) and *Trachelyopterus* (Ferraris, 1988) (unpublished thesis), whereas other authors recognized it as a valid genus (Akama, 2004) (unpublished thesis). To clarify the taxonomic status of ‘*Parauchenipterus*’, Akama (2004) (unpublished thesis) reviewed the group and proposed their recognition as distinct genera based on both external and internal morphological characters. However, a comprehensive phylogenetic study conducted by Calegari et al. (2019), integrating molecular and morphological data, subsequently concluded that the two genera are synonyms.

Although the synonymy between *Trachelyopterus* and *Parauchenipterus* has been resolved, several questions require further investigation, as some studies suggest that species diversity within *Trachelyopterus* is greater than currently recognized. For example, Akama (2004) proposed a putative new species, designated as *Trachelyopterus* sp. n., restricted to the Paraná‐Paraguay basin (Akama, 2004), which has not yet been formally described. This putative taxon has also been supported by cytogenetic evidence (referred to as *Trachelyopterus* aff. *coriaceus*; Haerter et al., 2022, 2023; Santos et al., 2021). Additionally, variation in cytogenetic markers has suggested the presence of another potential taxonomic unit in the Araguaia River basin (Haerter et al., 2022, 2023; Santos et al., 2021). Similarly, Birindelli et al. (2012) also showed morphological variation in the gas bladder of *Trachelyopterus galeatus* that may represent interspecific variation. Consequently, species diversity within *Trachelyopterus* is likely underestimated, particularly given the broad geographic distribution of the genus across South America. Therefore, the application of complementary approaches to investigate this diversity is essential for improving our understanding of biodiversity within the genus.

Therefore, considering the current taxonomic uncertainties within some species of *Trachelyopterus*, particularly regarding the potential occurrence of undescribed taxa, we investigated the genetic variation within *T. galeatus* and *T. coriaceus* using the DNA barcoding approach. Our main objectives were to clarify species boundaries and identify distinct lineages that may represent previously undescribed species.

## 2 MATERIALS AND METHODS

### 2.1 Collection and sampling sites

Our sampling included species from six hydrographic systems: the western Amazon, Araguaia‐Tocantins, Paraná, Paraguay, Suriname and coastal drainages. We sampled specimens of *T. galeatus* from Paraná, Amazonas and Araguaia‐Tocantins River basins; *T. coriaceus* from Araguaia‐Tocantins and Paraguay River basins; *T*. *striatulus* from coastal drainages and the Doce River basin; *T. porosus* from the Amazonas River basin (Figures [Link], [Link]); and one specimen from French Guiana, Kourou, biological station of Sokoumou, Kourou River (GenBank accession number MF595264.1; Calegari et al., 2019), the closest sample to the type locality (Suriname River) (for additional details on sampling locations, sample numbers and voucher and sequence data, see Table 1). Live specimens were collected with fish nets (SISBIO Permanent Licences 10538 and 49379) and euthanized by clove oil overdose (Griffiths, 2000; Lucena et al., 2013) (according to the Ethics Committee on Animal Experimentation: Protocol 13/09—CEEAAP/Unioeste). Tissue samples were obtained from liver cells and preserved in 99% ethanol. Voucher specimens were deposited at the Coleção Ictiológica do Núcleo de Pesquisa em Limnologia, Ictiologia e Aquicultura da Universidade Estadual de Maringá, Maringá (NUP); the Museu de Zoologia, Universidade de São Paulo, São Paulo (MZUSP); and the Instituto Nacional de Pesquisas da Amazônia, Manaus (INPA) (Table 1). The morphological identification of the specimens was performed by specialists in Auchenipteridae: Drs. Heraldo Antonio Britski, José Luís Olivan Birindelli and Jansen Alfredo Sampaio Zuanon.

**TABLE 1:** Taxa, sampling locations, number of specimens sampled, geographic coordinates and voucher numbers.

Species	Locality	HB	N	Coordinates	Voucher‐catalogue number	Genbank/BOLD
*Trachelyopterus galeatus*	Kourou River, Biological Station of Sokoumou	Sur	1	–	MHNG 2595.066/2608.013^a^	MF595264.1
*Trachelyopterus galeatus*	Jupiá Reservoir, Paraná River	Par	6	20°44′60,3″ S 51°39′10,5″ W	NUP8614	TRC044‐22 TRC045‐22 TRC046‐22 TRC047‐22 TRC048‐22 TRC049‐22
*Trachelyopterus galeatus*	Catalão Lake, confluence between Negro and Solimões Rivers	Amz	5	03°09′47″ S 59°54′29″ W	INPA 57,939	TRC050‐22 TRC051‐22 TRC052‐22 TRC053‐22 TRC054‐22
*Trachelyopterus* aff. *galeatus*	Marginal lake to the Medo stream, Ana Maria Farm	Ag	6	13°08′52,7″ S 50°25′02,8″ W	MZUSP 109,793	TRC055‐22 TRC056‐22 TRC057‐22 TRC058‐22 TRC059‐22 TRC060‐22
*Trachelyopterus porosus*	Catalão lake, confluence between Negro and Solimões Rivers	Amz	6	03°09′59,3″ S 59°58′02,5″ W	INPA 57,940	TRC061‐22 TRC062‐22 TRC063‐22 TRC064‐22 TRC065‐22 TRC066‐22
*Trachelyopterus striatulus*	Verde Lake, Doce River basin	CB‐Doce	6	19°49′44,5″ S 42°37′52,5″ W	MZUSP 109,798	TRC067‐22 TRC068‐22 TRC069‐22 TRC070‐22 TRC071‐22 TRC072‐22
*Trachelyopterus coriaceus*	Marginal lake to the Medo stream, Ana Maria Farm	Arag	6	13°08′52,7″ S 50°25′02,8″ W	MZUSP 106,766	TRC073‐22 TRC074‐22 TRC075‐22 TRC076‐22 TRC077‐22 TRC078‐22
*Trachelyopterus* aff. *coriaceus*	Arrombado pond, Bento Gomes River, tributary of the Cuiabá basin	Py	6	16°25′40,9″ S 56°25′07,4″ W	MZUSP 110,806	TRC079‐22 TRC080‐22 TRC081‐22 TRC082‐22 TRC083‐22 TRC084‐22

Abbreviations: AR, Araguaia River basin; BH, Hydrographic basin; CB‐Doce, Coastal Basin of the Southeast Atlantic (Doce River basin); N, number of specimens analysed; PG, Paraguay River basin; PR, Paraná River basin; SU, Suriname basin.

^a^ Specimen voucher.

## 3 MOLECULAR ANALYSES

### 3.1 DNA extraction, amplification and sequencing of cytochrome oxidase I *(COI)* gene fragments

DNA was extracted with phenol/chloroform/isoamyl alcohol following Sambrook et al. (2001). Total DNA concentration (ng/μL) was quantified on a 1% agarose gel using the Low DNA Mass Ladder marker (Invitrogen). DNA quantity was estimated by comparing the size and intensity of the marker band with that of the samples. A working DNA solution for routine analyses (50 ng/μL) was prepared by diluting the extracted DNA in polymerase chain reaction (PCR)‐grade water.

We amplified a fragment of approximately 650 bp of the (*COI*) gene from genomic DNA using the primers FISHF1 (5′ TCA ACC AAC CAC AAA GAC ATT GGC AC 3′) and FISHR1 (5′ TAG ACT TCT GGG TGG CCA AAG AAT CA 3′) described by Ward et al. (2005). PCR amplifications were performed for 41 individuals, including 17 specimens of *T. galeatus* (from three distinct localities), six of *T. porosus*, six of *T. striatulus* and 12 of *T. coriaceus*. PCRs were performed in a final volume of 25 μL containing 1X colourless PCR buffer, 1.3 mM MgCl_2_, 0.2 mM dNTP Mix (0.25 mM of each), 1 μM of each primer, 100 ng of genomic DNA, 1 U of Taq DNA polymerase and PCR‐grade water. The thermal cycling conditions consisted of an initial denaturation at 95°C for 120 s, followed by 36 cycles at 94°C for 30 s, 54°C for 30 s, 72°C for 60 s, and a final extension of 72°C for 10 min.

PCR amplicons were purified with polyethylene glycol (PEG) 8000 following Sambrook et al. (2001). Sequencing reactions were performed using the BigDye® terminator v3.1 cycle sequencing kit. The runs were made in 36 cm capillaries using the POP‐7 polymer. Sequencing was performed on an ABI 3730 DNA Analyser (Applied Biosystems), and sequence data were analysed using the Sequencing Analysis 5.3.1 software with the KB Base Caller.

### 3.2 Sequence alignment and molecular analyses

In this study, we generated 41 sequences representing four nominal species: 11 sequences of *T. galeatus* (from two distinct locations) and six sequences from individuals identified as *Trachelyopterus* aff. *galeatus*; six sequences of individuals identified as *T. porosus*; six sequences of *T. striatulus*; six sequences of *T. coriaceus*; and six sequences of individuals identified as *Trachelyopterus* aff. *coriaceus*. They were manually edited in MEGA X v10.0.5 (Kumar et al., 2018).

For the complete dataset, we also included one sequence of *T. galeatus* from the type locality, the Kourou River (Genbank ID: MF595264.1; Calegari et al., 2019) and three outgroup sequences: *Auchenipterichthys punctatus* (Valenciennes, 1840) (GenBank ID: MN460967, MN460966), *Trachelyopterichthys taeniatus* (Kner, 1858) (GenBank ID: MF595278.1) and *Trachelyichthys* sp. (GenBank ID: MF595203.1) (Table 1). The final alignment comprised 46 sequences representing seven valid species. Sequence alignment was performed using Clustal W (Larkin et al., 2007).

Genetic divergence, as well as intra‐ and interspecific distances, was calculated in MEGA X v10.0.5 (Kumar et al., 2018) using the Kimura 2‐parameter model (Kimura, 1980). Neighbour‐Joining (NJ)analysis was performed using the Tamura‐3‐parameter substitution model in MEGA X v10.0.5 (Kumar et al., 2018). Maximum Likelihood (ML) phylogenetic inference analyses were performed in MEGA X v10.0.5 (Kumar et al., 2018) using 1000 bootstrap replicates. Bayesian inference (BI) analyses were conducted in MrBayes v3.2.1 (Ronquist & Huelsenbeck, 2003) using two runs of four chains each (10 × 10^6^ generations), with trees sampled every 1000 generations, assuming a 25% burn‐in. The most appropriate nucleotide substitution model was Tamura‐3‐parameter+G according to the Akaike information criterion (*AIC*) in MEGA X v10.0.5. However, since MrBayes 3.1.2 implements only 1, 2 and 6 replacement templates, we applied the closest substitution model to minimize the potential effects of model violation or underparameterization (Li et al., 2007; McGuire et al., 2007). Accordingly, BI analyses were conducted using the following settings: ‘lset nst = 6 ‘(TN93), ‘rates = invgamma ‘(G + I). Branch support was estimated using posterior probabilities (BI).

Two species delimitation analyses were performed: assemble species by automatic partitioning (ASAP) analysis on the available web platform (https://bioinfo.mnhn.fr/abi/public/asap) (Puillandre et al., 2020) and unweighted pair group method with arithmetic mean (UPGMA) on MEGA X v10.0.5 (Kumar et al., 2018).

## 4 RESULTS

**FIGURE 1:**
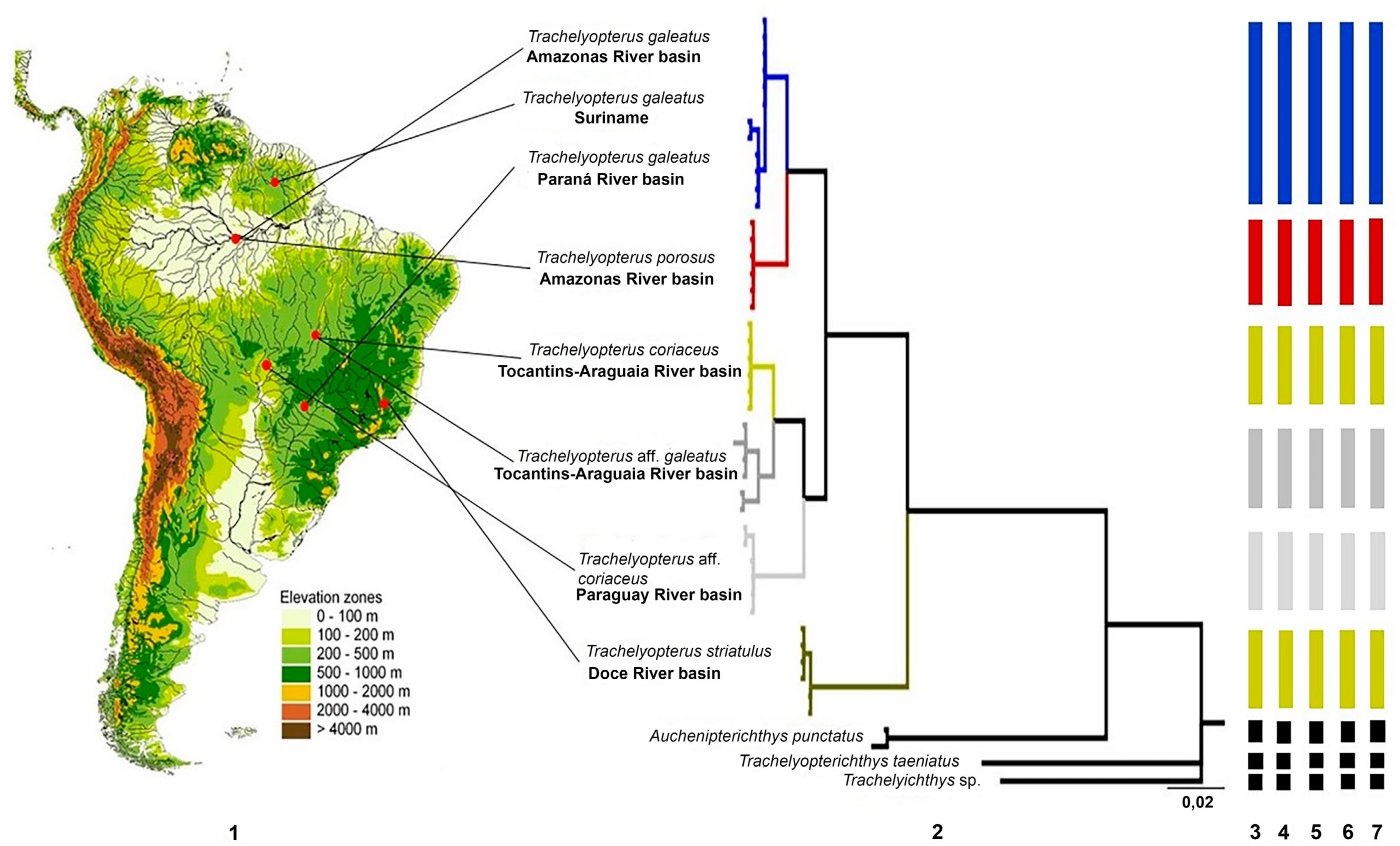
Sample collection points and results of *COI* sequence analyses from *Trachelyopterus* populations in comparison with previous morphological studies. (1) Map highlighting the collection localities of the populations/species used in this study; (2) phylogenetic representation inferred by Bayesian analysis, in which all nodes are supported by posterior probabilities of 100%; (3) maximum likelihood analysis results; (4) maximum Parsimony analyses; (5) neighbour‐joining analysis results; (6) unweighted pair group method with arithmetic mean (UPGMA) analysis results; (7) assemble species by automatic partitioning (ASAP) analysis results.

The final alignment comprised 607 base pairs (bp), including 77 polymorphic sites. The overall topology was consistent among the ML, NJ and BI analyses (Figures 1 and S8). Our analyses recovered six main clades: (a) *T. galeatus* samples from Paraná, Amazonas and Araguaia‐Tocantins River basins; (b) *T. galeatus* from the Araguaia‐Tocantins River basin; (c) *T. coriaceus* from the Araguaia‐Tocantins River basin; (d) *T. coriaceus* from the Paraguay River basin; (e) *T. striatulus*; and (f) *T. porosus*. Notably, the results did not support the monophyly of *T. galeatus* and *T. coriaceus*, as two lineages attributed to these taxa were recovered in distinct clades, rendering them paraphyletic. Considering the phylogenetic results, these lineages may represent distinct evolutionary units and should be further evaluated as potential separate species. In this manuscript, we refer to these lineages as *Trachelyopterus* aff. *galeatus* and *Trachelyopterus* aff. *coriaceus*, respectively. Species delimitation analyses (ASAP and UPGMA) yielded congruent results, recovering *Trachelyopterus* aff. *galeatus* and *Trachelyopterus* aff. *coriaceus* as distinct and independent lineages (Figure 1). The genetic distance between *Trachelyopterus* species and the outgroup taxa was approximately 21%.

Genetic distances among *T. galeatus* populations, including *Trachelyopterus* aff. *galeatus* ranged from 0.22% to 4.54%, with one pair of populations sharing identical haplotypes. The intragroup genetic distance of the combined group formed by *T. galeatus* from Suriname (Sur), Amazonas (Amz), Paraguay (Par) and *Trachelyopterus* aff. *galeatus* from the Araguaia‐Tocantins River basin (Sur + Amz + Par + Arag) was 2.1%. However, when the *Trachelyopterus* aff. *galeatus* population was excluded, and only specimens of *T. galeatus* from the Suriname, Amazonas and Paraná River basins (Sur + Amz + Par) were considered, the intragroup divergence decreased to 0.14% (Table 2). The *Trachelyopterus* aff. *galeatus* specimens from the Araguaia‐Tocantins River basin exhibited an intrapopulation genetic divergence of 0.5% (Table 2). Furthermore, these specimens showed greater genetic similarity to *T. coriaceus* (1.61%) than to the remaining *T*. *galeatus* populations (4.28% to 4.54%) (Table 2). This pattern was corroborated by both phylogenetic analyses and species delimitation analyses, which consistently recovered the Araguaia–Tocantins population as a distinct and independent lineage.

**TABLE 2:** Mean of interspecific and intraspecific genetic divergence calculated by the average distance using the Tamura three‐parameter model.

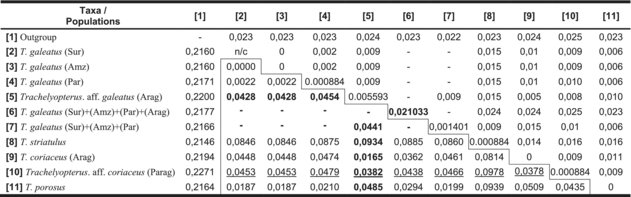

*Note*: Intraspecific variation values are shown on the diagonal of the table. Values highlighted in bold support *Trachelyopterus aff. galeatus* from the Araguaia River basin as a new species, whereas underlined values support *Trachelyopterus aff. coriaceus* from the Paraguay River basin as a new species [1] Outgroup; [2] *Trachelyopterus galeatus* (Suriname basin, Sur); [3] *Trachelyopterus galeatus* (Amazonas River basin, Amz); [4] *Trachelyopterus galeatus* (Paraná River basin, Par); [5] *Trachelyopterus* aff. *galeatus* (Araguaia‐Tocantins River basin, Arag); [6] *Trachelyopterus galeatus* (Suriname, Amazonas, Paraná and Araguaia‐Tocantins Rivers basin); [7] Group of *Trachelyopterus galeatus* (Suriname, Amazonas and Paraná Rivers basin); [8] *Trachelyopterus striatulus* (Doce River basin); [9] *Trachelyopterus coriaceus* (Araguaia‐Tocantins River basin); [10] *Trachelyopterus* aff. *coriaceus* (Paraguay River basin); [11] *Trachelyopterus porosus* (Amazonas River basin). N/C:not conclusive due to the presence of a single individual. Standard deviation up to1.5%.

*Trachelyopterus porosus* was the species most genetically similar to *T. galeatus*, with genetic distances ranging from 1.8% to 4.8%. In contrast, genetic distances between *T. porosus* and the remaining species included in this study ranged from 4.3% to 9.3% (Table 2). The genetic distance between the sympatric populations of *T. coriaceus* and *Trachelyopterus* aff. *galeatus* from the Araguaia‐Tocantins River basin was 1.6%. On the other hand, the genetic distance between *T. coriaceus* and the group composed of *T. galeatus* populations from Suriname, Amazonas and Paraguay (Sur + Amz + Par) was 4.6% (Table 2), decreasing to 3.6% when *Trachelyopterus* aff. *galeatus* from the Araguaia–Tocantins River basin was included in the analysis (Sur + Amz + Par + Arag). *Trachelyopterus striatulus* was the most genetically divergent species, exhibiting genetic distances ranging from 8.4% to 9.3% relative to all other taxa analysed.

Genetic distances between *Trachelyopterus* aff. *coriaceus* (Par) and *T. galeatus* populations, including *Trachelyopterus* aff. *galeatus*, ranged from 4.5% to 4.7%. Genetic distance between *Trachelyopterus* aff. *coriaceus* and *T. porosus* was 4.3%, whereas the distance to *T. coriaceus* was 3.7% (Table 2). Both the phylogenetic and species delimitation analyses recovered this population as a distinct and independent lineage.

## 5 DISCUSSION

The effectiveness of DNA barcoding for species identification relies on the presence of a clear difference between average within and between interspecific divergence, with interspecific divergence being at least 10 times greater and without overlap in the so‐called ‘barcode gap’ (Hebert et al., 2003; Hebert et al., 2004; Meier et al., 2006, 2008; Meyer & Paulay, 2005; Ward et al., 2005). In this study, we analysed four populations morphologically identified as *T. galeatus* (Suriname, Amazonas, Paraguay and Araguaia‐Tocantins River basins), one population of *T. striatulus* (Doce River basin), one population of *T. coriaceus* (Araguaia‐Tocantins River basin) and two populations of *T. porosus* (Amazonas and Paraguay River basins). Our results support the existence of two lineages that potentially represent undescribed species within *Trachelyopterus*: a population morphologically assigned to *T. galeatus* from the Araguaia‐Tocantins River basin (here referred to as *Trachelyopterus* aff. *galeatus*) and a population of *T. coriaceus* (here referred to as *Trachelyopterus* aff. *coriaceus*) from the Paraguay River.

*Trachelyopterus coriaceus* from the Araguaia River basin and *Trachelyopterus* aff. *coriaceus* from the Paraguay River basin exhibited very low intragroup variation (≤0.1%) when analysed separately (Table 2). However, the genetic distance between these two populations was 3.78% (Table 2), a value that exceeds by more than 10 times the commonly accepted threshold for species delimitation under the DNA barcoding method, supporting their status as distinct species. Although a ~2% divergence threshold is frequently used in DNA barcoding studies, this value is not universal, as several studies have reported species with lower levels of interspecific divergence. Therefore, such thresholds should be interpreted cautiously and used only as heuristic guidelines, together with additional evidence, including the evolutionary history and biological characteristics of the taxa under investigation (reviewed in Pereira et al., 2013).

Under the taxonomic framework proposed by Royero (1999) and Akama (2004), *Trachelyopterus* would include only two species, *Trachelyopterus coriaceus* Valenciennes, 1840, and *Trachelyopterus* sp. n., whereas the remaining taxa would be allocated to other genera. According to Akama (2004), *T. coriaceus* is distributed across the Amazonas, São Francisco and French Guiana Rivers, whereas *Trachelyopterus* sp. n. is restricted to the Paraná and Paraguay hydrographic basins. The *Trachelyopterus* aff. *coriaceus* population analysed in this study was collected from the Paraguay River basin, which is consistent with the geographic distribution previously proposed for *Trachelyopterus* sp. n. by Akama (2004). Furthermore, this identification was corroborated by the taxonomists responsible for examining the specimens included in this study. If these samples indeed represent the same lineage, our molecular results provide additional support for the recognition of *Trachelyopterus* sp. n. as a valid and distinct species that remains formally undescribed.

Currently, *T. galeatus* is widely distributed across South American river basins (Akama, 2004; Buckup et al., 2007; Calegari et al., 2019), a pattern that would generally be expected to result in levels of intraspecific genetic divergence. This expectation was partially supported by our results: when all populations of *T. galeatus* from Suriname, Amazonas, Paraguay and *Trachelyopterus* aff. *galeatus* were analysed as a single group, the intraspecific divergence exceeded 2%. However, when *Trachelyopterus* aff. *galeatus* was excluded, and only specimens of *T. galeatus* from the Suriname, Amazonas and Paraná river basins were considered, the intraspecific divergence decreased to 0.1% (Table 2). This result is particularly noteworthy given the large geographic distance separating these populations and the remarkably low intraspecific genetic variation observed among them after excluding the Tocantins‐Araguaia population. The populations from Catalão Lake (Amazon basin) and Jupiá Reservoir (Paraná River basin) exhibited low and null intraspecific genetic variation, respectively. Moreover, these populations showed a genetic divergence of only 0.22% and presented low or null divergence relative to the specimen from the type locality (Suriname) (Table 2). Based on these results, we confirm the identification of these samples as *T. galeatus*.

The genetic distance between the *Trachelyopterus* aff. *galeatus* and the remaining *T. galeatus* populations was approximately 12 times greater than the mean of intraspecific variation observed within *T. galeatus* and nearly six times higher than the divergence detected between the sympatric populations of *Trachelyopterus* aff. *galeatus* and T. *coriaceus*. These results support the hypothesis that *Trachelyopterus* aff. *galeatus* may represent an undescribed species. Similar interpretations have previously been suggested based on cytogenetic analyses of the same population (Haerter et al., 2022, 2023; Santos et al., 2021). The close relationship between *T. coriaceus* and *Trachelyopterus* aff. *galeatus* is further supported by cytogenetic evidence, which demonstrates greater similarity between them than with other *T. galeatus* populations or *Trachelyopterus* species (i.e., Araújo & Molina, 2013; Lui et al., 2009; Lui et al., 2010; Lui et al., 2021; Ravedutti & Júlio Jr., 2001; Santos et al., 2021).

A possible explanation for the phylogenetic placement of *Trachelyopterus* aff. *galeatus* is a historical hybridization event followed by introgression between ancestral lineages of *T. galeatus* (Arag) and *T. coriaceus*. In this study, each population was represented by six specimens (except for the specimen from the type locality), which is considered adequate for DNA barcoding analyses (Phillips et al., 2022). Our results revealed clear clustering of individuals within each taxon (without grouping specimens from the distinct sympatric populations). This pattern, in combination with the observed genetic distances, suggests a historical introgression event followed by geographical separation and the subsequent establishment of a secondary contact zone between these two lineages.

Mitochondrial introgression or incomplete lineage sorting may explain the similarity observed between mtDNA haplotypes of morphologically distinct species (McGuire et al., 2007). For example, Gaither et al. (2014) demonstrated that hybridization followed by introgression promoted mtDNA transfer among coral reef fishes, resulting in shared haplotypes across distinct species. Similarly, Decru et al. (2015) and Henriques et al. (2015) suggested that the inability of the *COI* sequence variation to discriminate certain species may reflect recent introgression events. Béres et al. (2017) investigated hybridization followed by introgression among *Ameiurus nebulosus* (Lesueur, 1819), *Ameiurus melas* (Rafinesque, 1820) and *Ameiurus natalis* (Lesueur, 1819) in Hungary that likely occurred two or more generations earlier. However, they employed additional genetic markers to support their hypothesis, an approach beyond the scope of the present study. Although these studies focus on relatively recent introgression events, evidence also exists for much older occurrences. For instance, Martin and Bermingham (2000), using both morphological and genetic data, investigated *Pimelodella chagresi* (Steindachner, 1876) from Central America and identified regional endemism, proposing that the species represents a cryptic species complex with a broad range of haplotypes potentially shaped by deterministic processes such as competitive exclusion or introgressive hybridization, particularly because the two most likely species within the complex occur in sympatry.

The putative introgression observed in *Trachelyopterus* aff. *galeatus* may have been facilitated by past geological events in the region, particularly the formation of the Araguaia Depression. This geological feature has been associated with the reactivation of extensive Precambrian continental fracture zones, leading to the development of vast floodplains that likely promoted continuous ichthyofaunal exchange among neighbouring drainage systems (Lima & Ribeiro, 2011; Saadi, 1993; Saadi et al., 2005). According to Saadi et al. (2005), the east of the Araguaia‐Tocantins Depression experienced gradual topographic uplift during the recent Cenozoic. This region also presents hydrological anomalies associated with ongoing tectonic activity, including the unresolved drainage systems known as ‘Seamed Waters’ (river headwaters coalescence systems). The interaction of recurrent geological and hydrological processes over time may have promoted both habitat fragmentation and secondary contact among populations of this group. This scenario provides a plausible explanation for the close genetic relationship observed between the sympatric populations of *T. galeatus* and *T. coriaceus*.

In this study, we identified six distinct lineages among the analysed samples: four currently recognized species [*T. galeatus* (Sur + Amz + Par), *T. coriaceus* (Arag), *T. porosus* (Amz) and *T. striatulus* (CB‐Doce)] and two putative undescribed species [*Trachelyopterus* aff. *galeatus* (Arag) and *Trachelyopterus* aff. *coriaceus* (Py)]. Notably, intrapopulation genetic divergences were either null or extremely low (≤0.1%) for most taxa, except for *Trachelyopterus* aff. *galeatus*, which exhibited a divergence of 0.5% (Table 2). Most taxa displayed intrapopulation divergence values below the average threshold proposed for species delimitation in fish (0.34%) (Ward, 2009). This low level of genetic variability reflects differences in the evolutionary histories and rates of diversification among them. Furthermore, most populations exhibiting low intraspecific variation in our study were sampled in ponds, where inbreeding is an important factor potentially accounting for the reduced genetic diversity observed.

The influence of genetic drift on these populations cannot be ruled out, particularly considering their occurrence in small and isolated ponds, conditions that increase susceptibility to stochastic effects relative to more widely distributed populations. As noted by Matsubara et al. (2001), locally fragmented populations may diverge through genetic drift when isolation is maintained; however, if gene flow persists among subpopulations, they may remain connected under a metapopulation dynamic. It is important to emphasize, however, that *T. galeatus* (Sur + Amaz+Par) exhibited an intraspecific variation of only 0.14% despite its broad geographic distribution. Therefore, another possible explanation for the low intraspecific variation observed is that it may represent an intrinsic characteristic of this group, potentially reflecting a relatively recent evolutionary origin.

## 6 CONCLUSION

Our results reinforce the utility of DNA barcoding for species detection and delimitation. This approach successfully revealed distinct biological lineages within what is currently recognized as *T. galeatus* and *T. coriaceus*, supporting the hypothesis that these taxa may represent a species complex. Moreover, our findings corroborate the existence of a putative new taxon previously proposed by Akama (2004) as *Trachelyopterus* sp. n. (here referred to as *Trachelyopterus* aff. *coriaceus*). Therefore, the identification key for *Trachelyopterus* should be reassessed and updated. A more comprehensive understanding of the relationships within the genus will require broader sampling, including a greater number of specimens, wider geographic coverage, and particular attention to potential cryptic diversity. In addition, the incorporation of additional molecular markers will be essential for accurately characterizing the diversity within *Trachelyopterus*, as our results demonstrate that morphological characters alone are insufficient for reliably delimiting species boundaries.

## AUTHOR CONTRIBUTIONS

Fiorindo José Cerqueira, Vladimir Pavan Margarido, Daniel Rodrigues Blanco and Roberto Laridondo Lui conceived the study; Fiorindo José Cerqueira conducted the experiments; Chrystian Aparecido Grillo Haerter, Vladimir Pavan Margarido, Sandra Mariotto, Liano Centofante, Maelin da Silva, and Roberto Laridondo Lui analysed the data; Eliana Feldberg, Vladimir Pavan Margarido, Orlando Moreira Filho and Roberto Laridondo Lui provided infrastructure and resources; and Fiorindo José Cerqueira and Chrystian Aparecido Grillo Haerter wrote the original draft. All authors read and approved the final version.

## FUNDING INFORMATION

This study was funded by the Fundação Araucária de Apoio ao Desenvolvimento Científico e Tecnológico do Paraná (FAPPR), Fundação de Amparo à Pesquisa do Estado de São Paulo (FAPESP), Coordenação de Aperfeiçoamento de Pessoal de Nível Superior (CAPES) and Conselho Nacional de Desenvolvimento Científico e Tecnológico (CNPq).

## Supporting information


**Figure S1.** Preserved specimen of *Trachelyopterus galeatus* from Catalão Lake, confluence between the Negro and Solimões Rivers, Amazonas River basin (INPA‐57939).

**Figure S2.** Preserved specimen of *Trachelyopterus porosus* from Catalão Lake, confluence between the Negro and Solimões Rivers, Amazonas River basin (INPA‐57940).

**Figure S3.** Preserved specimen of *Trachelyopterus coriaceus* from Marginal Lake to the Medo stream, Ana Maria Farm, Araguaia River basin (MZUSP106766).

**Figure S4.** Preserved specimen of *Trachelyopterus galeatus* from Marginal Lake to the Medo stream, Ana Maria Farm, Araguaia River basin (MZUSP109793).

**Figure S5.** Preserved specimen of *Trachelyopterus galeatus* from the Jupiá Reservoir, Paraná River (NUP‐8614).

**Figure S6.** Preserved specimen of *Trachelyopterus striatulus* from the Verde Lake, Doce River basin (MZUSP109798).

**Figure S7.** Preserved specimen of *Trachelyopterus* aff. *coriaceus* from the Arrombado pond, Bento Gomes River, tributary of the Cuiabá River basin, Paraguay River basin (MZUSP110806).

**Figure S8.** Phylogenetic representation of *Trachelyopterus* species used in this study and inferred by Bayesian analysis using the *COI* sequence.

## Data Availability

The data that support the findings of this study are available within the article and its supplementary files. The sequences used in this study are publicly available at the GenBank/BOLD databases.

## References

[jfb70540-bib-0001] Akama, A. (2004). Systematics of genus Parauchenipterus Bleeker, 1862 and Trachelyopterus Valenciennes, 1840 (Siluriformes, Auchenipteridae) (Doctoral thesis, University of São Paulo, Brazil).

[jfb70540-bib-0002] Albert, J. S. , & Reis, R. E. (2011). Introduction to neotropical freshwaters. In J. S. Albert & R. E. Reis (Eds.), Historical biogeography of Neotropical freshwater fishes (pp. 3–19). University of California Press.

[jfb70540-bib-0003] Araújo, W. C. , & Molina, W. F. (2013). Citótipo exclusivo para *Parauchenipterus galeatus* (Siluriformes, Auchenipteridae) na bacia do Atlântico NE Oriental do Brasil: indicações de um complexo de espécies. Biota Amazônia, 3, 33–39.

[jfb70540-bib-0004] Béres, B. , Kainain Sipos, D. , Müller, T. , Fazekas, G. , Specziár, A. , & Boros, G. (2017). Species‐specific markers provide molecular genetic evidence for natural introgression of bullhead catfishes in Hungary. PeerJ, 5, e2804. 10.7717/peerj.2804 PMC5333548 28265489

[jfb70540-bib-0005] Birindelli, J. L. O. (2014). Phylogenetic relationships of the south American Doradoidea (Ostariophysi: Siluriformes). Neotropical Ichthyology, 12, 451–564.

[jfb70540-bib-0006] Birindelli, J. L. O. , Akama, A. , & Britski, H. A. (2012). Comparative morphology of the gas bladder in driftwood catfishes (Siluriformes: Auchenipteridae). Journal of Morphology, 273, 651–660. 10.1002/jmor.20012 22290561

[jfb70540-bib-0007] Britski, H. A. (1972). Systematics and evolution of Auchenipteridae and Ageneiosidae (Teleostei, Siluriformes) (Doctoral thesis, University of São Paulo, Brazil).

[jfb70540-bib-0008] Buckup, P. A. , Menezes, N. A. , & Ghazzi, M. S. (2007). Catalog of freshwater fish species in Brazil (Vol. 23, pp. 1–16). National Museum of the Federal University of Rio de Janeiro.

[jfb70540-bib-0009] Calegari, B. B. , Vari, R. P. , & Reis, R. E. (2019). Phylogenetic systematics of the driftwood catfishes (Siluriformes: Auchenipteridae): A combined morphological and molecular analysis. Zoological Journal of the Linnean Society, 187, 661–773.

[jfb70540-bib-0010] Cooke, G. M. , Chao, N. L. , & Beheregaray, L. B. (2012). Five cryptic species in the Amazonian catfish *Centromochlus existimatus* identified based on biogeographic predictions and genetic data. PLoS One, 7(11), e48800. 10.1371/journal.pone.0048800 PMC3492235 23144977

[jfb70540-bib-0011] Curran, D. J. (1989). Phylogenetic relationships among the catfish genera of the family Auchenipteridae (Teleostei: Siluroidea). Copeia, 1989(2), 408–419.

[jfb70540-bib-0012] Dagosta, F. C. P. , & Albert, J. S. (2024). Fishes of the upper Rio Paraná basin: Diversity, biogeography and conservation. Neotropical Ichthyology, 22, e230066.

[jfb70540-bib-0013] Dagosta, F. C. P. , & de Pinna, M. C. C. (2019). The fishes of the Amazon: Distribution and biogeographical patterns, with a comprehensive list of species. Bulletin of the American Museum of Natural History, 431, 1–163.

[jfb70540-bib-0014] Decru, E. , Moelants, T. , Van den Berghe, E. , Vreven, E. , Verheyen, E. , & Snoeks, J. (2015). Taxonomic challenges in freshwater fishes: A mismatch between morphology and DNA barcoding in fish of the north‐eastern part of The Congo basin. Molecular Ecology Resources, 16, 342–352. 10.1111/1755-0998.12445 26186077

[jfb70540-bib-0015] Eigenmann, C. H. , & Eigenmann, R. S. (1888). Preliminary notes on south American Nematognathi. Proceedings of the California Academy of Sciences, 1(2), 119–172.

[jfb70540-bib-0016] Eigenmann, C. H. , & Eigenmann, R. S. (1890). A revision of the south American Nematognathi, or catfishes. Occasional Papers of the California Academy of Sciences, 1, 1–508.

[jfb70540-bib-0017] Fernandes, F. M. C. , Albert, J. S. , Daniel‐Silva, M. D. Z. , Alves‐Gomes, J. A. , Triques, M. , & Maldonado‐Ocampo, J. A. (2005). A new *Gymnotus* (Teleostei: Gymnotiformes: Gymnotidae) from the Pantanal Matogrossense of Brazil and adjacent drainages: Continued documentation of a cryptic fauna. Zootaxa, 933, 1–14.

[jfb70540-bib-0018] Ferraris, C. J. J. (1988). The Auchenipteridae: Putative monophyly and systematics, with a classification of the Neotropical Doradoid catfishes (Ostariophysi: Siluriformes) (Doctoral thesis, University of New York, USA).

[jfb70540-bib-0019] Gaither, M. R. , Schultz, J. K. , Bellwood, D. R. , & Bowen, B. W. (2014). Evolution of pygmy angelfishes: Recent divergences, introgression, and the usefulness of color in taxonomy. Molecular Phylogenetics and Evolution, 74, 38–47. 10.1016/j.ympev.2014.01.017 24500654

[jfb70540-bib-0020] Griffiths, S. P. (2000). The use of clove oil as an anaesthetic and method for sampling intertidal rockpool fishes. Journal of Fish Biology, 57, 1453–1464.

[jfb70540-bib-0021] Haerter, C. A. G. , Blanco, D. R. , Traldi, J. B. , Feldberg, E. , Margarido, V. P. , & Lui, R. L. (2023). Are scattered microsatellites weak chromosomal markers? Guided mapping reveals new insights into *Trachelyopterus* (Siluriformes: Auchenipteridae) diversity. PLoS One, 18, e0285388. 10.1371/journal.pone.0285388 PMC10263338 37310952

[jfb70540-bib-0022] Haerter, C. A. G. , Margarido, V. P. , Blanco, D. R. , Traldi, J. B. , Feldberg, E. , & Lui, R. L. (2022). Contributions to *Trachelyopterus* (Siluriformes: Auchenipteridae) species diagnosis by cytotaxonomic autapomorphies: From U2 snRNA chromosome polymorphism to rDNA and histone gene synteny. Organisms Diversity & Evolution, 22, 1021–1036.

[jfb70540-bib-0023] Hebert, P. D. N. , Cywinska, A. , Ball, S. L. , & deWaard, J. R. (2003). Biological identifications through DNA barcodes. Proceedings of the Royal Society B: Biological Sciences, 270, 313–321. 10.1098/rspb.2002.2218 PMC1691236 12614582

[jfb70540-bib-0024] Hebert, P. D. N. , Penton, E. H. , Burns, J. M. , Janzen, D. H. , & Hallwachs, W. (2004). Ten species in one: DNA barcoding reveals cryptic species in the neotropical skipper butterfly *Astraptes fulgerator* . Proceedings of the National Academy of Sciences of the United States of America, 101, 14812–14817. 10.1073/pnas.0406166101 PMC522015 15465915

[jfb70540-bib-0025] Henriques, R. , van der Heyden, S. , & Matthee, C. A. (2015). When homoplasy mimics hybridization: A case study of cape hakes (*Merluccius capensis* and *M. paradoxus*). PeerJ, 4, e1827. 10.7717/peerj.1827 PMC4824878 27069785

[jfb70540-bib-0026] Kimura, M. (1980). A simple method for estimating evolutionary rate of base substitutions through comparative studies of nucleotide sequences. Journal of Molecular Evolution, 16, 111–120. 10.1007/BF01731581 7463489

[jfb70540-bib-0027] Kumar, S. , Stecher, G. , Li, M. , Knyaz, C. , & Tamura, K. (2018). MEGA X: Molecular evolutionary genetics analysis across computing platforms. Molecular Biology and Evolution, 35, 1547–1549. 10.1093/molbev/msy096 PMC5967553 29722887

[jfb70540-bib-0028] Larkin, M. A. , Blackshields, G. , Brown, N. P. , Chenna, R. , McGettigan, P. A. , McWilliam, H. , Valentin, F. , Wallace, I. M. , Wilm, A. , Lopez, R. , Thompson, J. D. , Gibson, T. J. , & Higgins, D. G. (2007). Clustal W and Clustal X version 2.0. Bioinformatics, 23, 2947–2948. 10.1093/bioinformatics/btm404 17846036

[jfb70540-bib-0029] Li, C. , Ortí, G. , Zhang, G. , Lu, G. , & Wen, Z. (2007). A practical approach to phylogenomics: The phylogeny of ray‐finned fish (Actinopterygii) as a case study. BMC Evolutionary Biology, 7, 44. 10.1186/1471-2148-7-44 PMC1838417 17374158

[jfb70540-bib-0030] Lima, F. C. T. , & Ribeiro, A. C. (2011). Continental‐scale tectonic controls of biogeography and ecology. In J. S. Albert & R. E. Reis (Eds.), Historical biogeography of Neotropical freshwater fishes. University of California Press.

[jfb70540-bib-0031] Lucena, C. A. , Calegari, B. , Pereira, E. , Britski, H. A. , & Pavanelli, C. S. (2013). O uso de óleo de cravo na eutanásia de peixes. Boletim da Sociedade Brasileira de Ictiologia, 105, 20–24.

[jfb70540-bib-0032] Lui, R. L. , Blanco, D. R. , Margarido, V. P. , & Moreira‐Filho, O. (2009). First description of B chromosomes in the family Auchenipteridae, *Parauchenipterus galeatus* (Siluriformes) of the São Francisco River basin (MG, Brazil). Micron, 40, 552–559. 10.1016/j.micron.2009.03.004 19394233

[jfb70540-bib-0033] Lui, R. L. , Blanco, D. R. , Margarido, V. P. , & Moreira‐Filho, O. (2010). Chromosome characterization and biogeographic relations among three populations of the driftwood catfish *Parauchenipterus galeatus* (Linnaeus, 1766) (Siluriformes: Auchenipteridae) in Brazil. Biological Journal of the Linnean Society, 99, 648–656.

[jfb70540-bib-0034] Lui, R. L. , Traldi, J. B. , Blanco, D. R. , Margarido, V. P. , Mariotto, S. , Centofante, L. , Artoni, R. F. , & Moreira‐Filho, O. (2021). Possible common origin of B chromosomes in neotropical fish (Siluriformes: Auchenipteridae) reinforced by repetitive DNA mapping. Brazilian Archives of Biology and Technology, 64, e21190494.

[jfb70540-bib-0035] Martin, A. P. , & Bermingham, E. (2000). Regional endemism and cryptic species revealed by molecular and morphological analysis of a widespread species of Neotropical catfish. Proceedings of the Royal Society of London B, 267, 1135–1141. 10.1098/rspb.2000.1119 PMC1690641 10885519

[jfb70540-bib-0036] Matsubara, H. , Sakai, H. , & Iwata, A. (2001). A river metapopulation structure of a Japanese freshwater goby, *Odontobutis obscura*, deduced from allozyme genetic indices. Environmental Biology of Fishes, 61, 285–294.

[jfb70540-bib-0037] McGuire, J. A. , Linkem, C. W. , Koo, M. S. , Lemmon, A. R. , & Lemmon, E. M. (2007). Mitochondrial introgression and incomplete lineage sorting through space and time: Phylogenetics of crotaphytid lizards. Evolution, 61, 2879–2897. 10.1111/j.1558-5646.2007.00239.x 17941840

[jfb70540-bib-0038] Mees, G. F. (1974). The Auchenipteridae and Pimelodidae of Suriname (Pisces, Nematognathi). Zoologische Verhandelingen, 132, 1–256.

[jfb70540-bib-0039] Meier, R. , Kwong, S. , Vaidya, G. , & Ng, P. K. L. (2006). DNA barcoding and taxonomy in Diptera: A tale of high intraspecific variability and low identification success. Systematic Biology, 55, 715–728. 10.1080/10635150600969864 17060194

[jfb70540-bib-0040] Meier, R. , Zhang, G. , & Ali, F. (2008). The use of mean instead of smallest interspecific distances exaggerates the size of the “barcoding gap” and leads to misidentification. Systematic Biology, 57, 809–813. 10.1080/10635150802406343 18853366

[jfb70540-bib-0041] Meyer, C. P. , & Paulay, G. (2005). DNA barcoding: Error rates based on comprehensive sampling. PLoS Biology, 3, e422. 10.1371/journal.pbio.0030422 PMC1287506 16336051

[jfb70540-bib-0042] Nagamachi, C. Y. , Pieczarka, J. C. , Milhomem, S. S. R. , Silva, D. M. Z. , Nagamachi, F. N. , & Cioffi, M. B. (2010). Multiple rearrangements in cryptic species of electric knifefish, *Gymnotus carapo* (Gymnotidae, Gymnotiformes) revealed by chromosome painting. BMC Genetics, 11, 28. 10.1186/1471-2156-11-28 PMC2873553 20420709

[jfb70540-bib-0044] Pereira, L. H. , Hanner, R. , Foresti, F. , & Oliveira, C. (2013). Can DNA barcoding accurately discriminate megadiverse Neotropical freshwater fish fauna? BMC Genetics, 14, 20. 10.1186/1471-2156-14-20 PMC3608943 23497346

[jfb70540-bib-0045] Phillips, J. D. , Gillis, D. J. , & Hanner, R. H. (2022). Lack of statistical rigor in DNA barcoding likely invalidates the presence of a true species' barcode gap. Frontiers in Ecology and Evolution, 10, 859099.

[jfb70540-bib-0046] Puillandre, N. , Brouillet, S. , & Achaz, G. (2020). ASAP: Assemble species by automatic partitioning. Molecular Ecology Resources, 21(2), 609–620. 10.1111/1755-0998.13281 33058550

[jfb70540-bib-0047] Ravedutti, C. G. , & Júlio, H. F., Jr. (2001). Cytogenetic analysis of three species of the neotropical family Auchenipteridae (Pisces: Siluriformes) from the Paraná River Basin, Brazil. Cytologia, 66, 65–70.

[jfb70540-bib-0048] Regan, C. T. (1911). The classification of the teleostean fishes of the order Ostaripophysi. 2. Siluroidea. Annals and Magazine of Natural History, 8, 552–577.

[jfb70540-bib-0049] Reis, R. E. , Albert, J. S. , di Dario, F. , Mincarone, M. M. , Petry, P. , & Rocha, L. A. (2016). Fish biodiversity and conservation in South America. Journal of Fish Biology, 89, 12–47. 10.1111/jfb.13016 27312713

[jfb70540-bib-0051] Ronquist, F. , & Huelsenbeck, J. P. (2003). MrBayes 3: Bayesian phylogenetic inference under mixed models. Bioinformatics, 19, 1572–1574. 10.1093/bioinformatics/btg180 12912839

[jfb70540-bib-0052] Royero, R. L. (1999). Studies on the systematics and phylogeny of the catfish family Auchenipteridae (Teleostei: Siluriformes). Ph.D. Dissertation, University of Bristol.

[jfb70540-bib-0053] Saadi, A. (1993). Neotectônica da plataforma brasileira: esboço e interpretação preliminares. Geonomos, 1, 1–15.

[jfb70540-bib-0054] Saadi, A. , Bezerra, F. H. R. , Costa, R. D. , Soares‐Silva, L. M. , Suguio, K. , & Souza, C. R. G. (2005). Neotectônica da Plataforma Brasileira. In C. R. G. Souza , K. Suguio , A. M. S. Oliveira , & P. E. Oliveira (Eds.), Quaternário do Brasil. Ribeirão Preto.

[jfb70540-bib-0055] Sambrook, J. , Fritsch, E. F. , & Maniatis, T. (2001). Molecular cloning: A laboratory manual (3rd ed.). Cold Spring Harbor Laboratory Press.

[jfb70540-bib-0056] Santos, D. P. , Felicetti, D. , Baumgärtner, L. , Moreira‐Filho, O. , Silva, D. M. Z. , & Cioffi, M. B. (2021). Contributions to the taxonomy of *Trachelyopterus* (Siluriformes): Comparative cytogenetic analysis in three species of Auchenipteridae. Neotropical Ichthyology, 19, e200115.

[jfb70540-bib-0057] Tedesco, P. A. , Beauchard, O. , Bigorne, R. , Cornu, J. F. , Daget, J. , de Wever, A. , Dürr, H. H. , Leprieur, F. , & Brosse, S. (2017). A global database on freshwater fish species occurrence in drainage basins. Scientific Data, 4, 170141. 10.1038/sdata.2017.141 PMC5625552 28972575

[jfb70540-bib-0058] Valentini, A. , Pompanon, F. , & Taberlet, P. (2008). DNA barcode for ecologists. Trends in Ecology & Evolution, 24, 110–116. 10.1016/j.tree.2008.09.011 19100655

[jfb70540-bib-0059] Ward, R. D. (2009). DNA barcode divergence among species and genera of birds and fishes. Molecular Ecology Resources, 9, 1077–1085. 10.1111/j.1755-0998.2009.02541.x 21564845

[jfb70540-bib-0060] Ward, R. D. , Zemlak, T. S. , Innes, B. R. , Last, P. R. , & Hebert, P. D. N. (2005). DNA barcoding Australia's fish species. Philosophical Transactions of the Royal Society B: Biological Sciences, 360, 1847–1857. 10.1098/rstb.2005.1716 PMC1609232 16214743

